# Does the Mediterranean Diet Protect against Stress-Induced Inflammatory Activation in European Adolescents? The HELENA Study

**DOI:** 10.3390/nu10111770

**Published:** 2018-11-15

**Authors:** Kenia M. B. Carvalho, Débora B. Ronca, Nathalie Michels, Inge Huybrechts, Magdalena Cuenca-Garcia, Ascensión Marcos, Dénes Molnár, Jean Dallongeville, Yannis Manios, Beatriz D. Schaan, Luis Moreno, Stefaan de Henauw, Livia A. Carvalho

**Affiliations:** 1 Graduate Program in Human Nutrition, University of Brasília, Brasília 70910-900, Brazil; deboraronca@gmail.com; 2 Department of Public Health, Faculty of Medicine and Health Sciences, Ghent University, 9000 Ghent, Belgium; Nathalie.Michels@UGent.be (N.M.); HuybrechtsI@iarc.fr (I.H.); stefaan.dehenauw@ugent.be (S.d.H.); 3 International Agency for Research on Cancer, 69372 Lyon, France; 4 Department of Medical Physiology, School of Medicine, University of Granada, 18071 Granada, Spain; magdalena.cuenca@uca.es; 5 ICTAN-CSIC Spanish National Research Council, 28040 Madrid, Spain; amarcos@ictan.csic.es; 6 Department of Paediatrics, Medical School, University of Pécs, 7623 Pécs, Hungary; denes.molnar@aok.pte.hu; 7 Institut Pasteur de Lille, 59800 Lille, France; jean.dallongeville@pasteur-lille.fr; 8 Department of Nutrition and Dietetics, Harokopio University, 17671 Athens, Greece; helena@hua.gr; 9 Graduate Program in Medical-Sciences, Endocrinology, Federal University of Rio Grande do Sul, Porto Alegre 90035-003, Brazil; bschaan@hcpa.edu.br; 10 GENUD (Growth, Exercise, Nutrition and Development) Research Group, 50013 Zaragoza, Spain; lmoreno@unizar.es; 11 Department of Clinical Pharmacology, William Harvey Research Institute, Barts and The London Hospital, Queen Mary University of London, London E1 4NS, UK; l.carvalho@qmul.ac.uk

**Keywords:** diet quality, depressive symptoms, risk factors, epidemiology, immune system, prevention, hypothalamic–pituitary–adrenal-HPA axis

## Abstract

Stress increases inflammation but whether adherence to Mediterranean diet counteracts this association and how early can these effects be observed is not well known. We tested whether (1) cortisol is associated to inflammation, (2) cortisol is associated to the adolescent Mediterranean diet score (aMDS), (3) aMDS lessens inflammation, (4) aMDS associates with cortisol levels and inflammation. Two hundred and forty-two adolescents (137 females; 12.5–17.5 years old) provided salivary cortisol, blood and 2-day 24-h dietary recall from which aMDS was derived. Cortisol levels were associated with increased tumor necrosis factor (TNF-α *B* = 11.887, *p* = 0.001) when adjusted for age, gender, parental education and body mass index (BMI). Moreover, cortisol levels were inversely associated to adherence to the Mediterranean Diet (*B* = −1.023, *p* = 0.002). Adolescents with higher adherence to aMDS had lower levels of interleukins (IL) IL-1, IL-2, IL-6 and TNF-α, compared to those who did not adhere. The association between cortisol and TNF-α was no longer significant when aMDS was included in the model (*B* = 6.118, *p* = 0.139). In addition, comparing lower and higher aMDS groups, the association between cortisol and TNF-α was only observed in those with lower aMDS adherence. Our study suggests that adherence to the Mediterranean Diet may counteract the effect of stress on inflammatory biomarkers which may contribute to decreasing the risk of future mental health.

## 1. Introduction

During adolescence significant physical, emotional and physiological changes occur [1]. Stress may contribute towards unhealthy behaviors that poses mental health risk [2]. Approximately half of all psychiatric disorders start between late adolescence and early adulthood and predicts future psychopathology in adulthood [3,4,5]. There is now an extensive body of data showing that depression or stress are associated with a chronic low-grade inflammation [6,7]. Elevated levels of pro-inflammatory cytokines are observed in children and adolescents with major depressive disorder [8,9,10].

Diet is a modifiable behavior that impact levels of systemic inflammation [11,12,13,14]. The decreased systemic inflammation due to Mediterranean Diet can already be observed in adolescents [15]. Although not consistently observed [16], a systematic review shows an association between unhealthy diet with mental health disorders. In addition, an inflammatory dietary pattern based on lower levels of poly-saturated fatty acids (PUFA) n3 and higher levels of n6 is associated with risk of future depression [17]. Adherence to the Mediterranean diet attenuates the unfavorable effect of depression and anxiety on cardiovascular risk [18]. A healthy diet also protects the progression of inflammation and depression in the elderly [19]. In adolescents, better diet quality is also associated to depressed mood [20,21] but whether this is due to counteracting stress-induced inflammation is not yet known.

The aim of this study in adolescents was to test whether: 1—cortisol is associated with inflammation, 2—cortisol is associated with Mediterranean diet adherence, 3—Mediterranean diet adherence lessens inflammation, 4—Mediterranean diet adherence associates with cortisol biomarker and inflammation.

## 2. Materials and Methods

### 2.1. Study Design and Population

**Figure 1:**
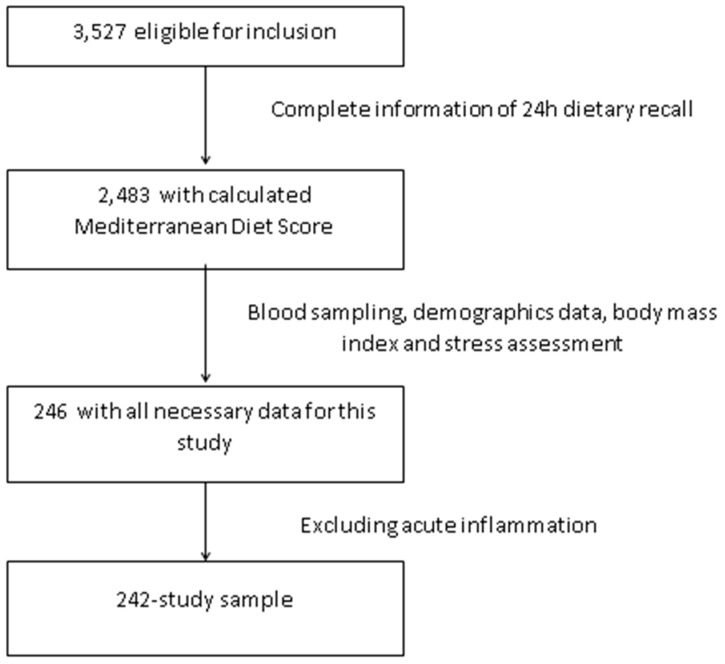
Flow chart of the selection procedure of the study sample among participants of Healthy Lifestyle in Europe by Nutrition in Adolescence Cross-Sectional Study (HELENA-CSS.)

The HELENA-CSS (Healthy Lifestyle in Europe by Nutrition in Adolescence Cross-Sectional Study) is a multi-center study aiming to obtain reliable and comparable data on a broad variety of parameters related to nutrition and health in European adolescents [22]. The methodology used in this study has been published elsewhere [23]. Briefly, subjects aged 12.5–17.5 years were recruited from schools of 10 cities from nine countries across Europe (Greece, Germany, Belgium, France, Hungary, Italy, Sweden, Austria and Spain). Inclusion criteria included not participating simultaneously in any clinical trial, being free of any acute infection occurring within the week prior to the study and data being available concerning an individual’s gender, height and weight. The enrollment of adolescents occurred at schools where students from the first two randomly chosen classes were invited to participate. In addition, the class attendance rate of at least 70% was considered for eligibility. The total eligible population consisted of 3528 adolescents. Blood and salivary sample was only collected in a random third of the population study. We have analyzed everyone who had complete data for the exposure, outcome and covariates used. Thus, we had complete data for 246 adolescents on diet, inflammatory markers and cortisol. We excluded subjects with C-reactive protein (CRP) >10 mg/L as a sign of acute infection (*n* = 4) so that the final sample consisted of 242 adolescents (Figure 1). In comparison to the total eligible HELENA-CSS population (*n* = 3528), this sample was less overweight and obese (14.0% vs. 23.9%, *p* < 0.001), had higher adherence to the Mediterranean diet (45.0% vs. 37.3%, *p* = 0.019), higher level of parental education (56.6% vs. 43.6% University degree, *p* < 0.001) but no differences in age (*p* = 0.916) or gender (*p* = 0.164).

### 2.2. Procedures

The Research Ethics Committees of each center involved approved the study protocol. Written informed consent was obtained from adolescents and their parents [24]. The fieldwork was carried out from October 2006 to December 2007 and consisted of clinical examination, blood and saliva sampling, questionnaires and dietary intake assessment [22]. All data was collected on the same day as the blood and saliva samples, measures needing repeat collection were conducted on the same week.

### 2.3. Measurements

#### 2.3.1. Pubertal Stage, Nutritional Status and Socio-Demographics Characteristics

During the clinical examination, pubertal stage was assessed according to development of secondary sexual characteristics (breast/genitalia and pubic hair development) and using the five stages of development devised by Marshall and Tanner [25,26]. Body weight was determined to the nearest 100 g using a scale (SECA 861) with subjects in their underwear. Height was assessed to the nearest 0.1 cm with a stadiometer (SECA 225) while standing barefoot [27]. Body mass index (BMI) was calculated as weight (kg) divided by height (m) squared. The corresponding BMI z-score was calculated with reference to sex and age parameters and categorized in corresponding nutritional status [28]. BMI was coded (i) normal/underweight and (ii) overweight/obese [28]. The highest parental educational achievement was used and categorized in (i) lower education/higher secondary and (ii) University degree.

#### 2.3.2. Salivary Cortisol Biomarkers

Baseline wake-up salivary free cortisol was measured in the adolescents as a biomarker for chronic stress according to previously published method [29]. Cortisol was measured in the accredited routine laboratory of Ghent University hospital on a Modular E 170 immunoanalyzer system (Roche Diagnostics, Mannheim, Germany) by the Roche Cobas Cortisol assay. The precise working mechanism and features of this analysis technique are described elsewhere [30]. This competitive electrochemiluminescence immunoassay had an inter-assay coefficient of variation of 3.9% and an intra-assay coefficient of variation of 1.9%, while for samples near the lower detection limit the coefficients of variation were respectively 12.7 and 10.2% (based on laboratory’s internal quality assessment). Cortisol is the main hormonal end-product of the ‘stress system.’ Serum cortisol is the result of appraising all stress-inputs on the brain, coping and recovery from them and are influenced by several neuro-endocrine and physiological pathways. Unbound or free cortisol is also present in the saliva and is positively associated with acute and chronic stress. To reduce variance in cortisol biomarkers due to diurnal variations in salivary cortisol, baseline (without stimulation) salivary cortisol was measured immediately after awakening. In order to control individual variability in salivary cortisol, awakening samples from seven consecutive days were collected. Saliva was sampled during the same week as the inflammatory markers.

#### 2.3.3. Inflammatory Markers

Blood samples were collected early morning after overnight fasting. For analysis of serum proteins, blood was collected in Vacutainer™ tubes (BD Biosciences, San Jose, CA, USA). Within the hour, serum was separated by centrifugation at 3500 rpm for 15 min; aliquots were made and sent to Bonn (Germany) in cooled containers on the same day and stored at −80 °C. At the end of the study, all samples were sent to Madrid (Spain) on dry ice and stored at −80 °C until analysis. The handling and transport system for fresh blood samples developed for the HELENA study assured stability of markers included in the analyses [29]. Serum levels of interleukin (IL)-1, IL-2, IL-4, IL-6 and tumor necrosis factor-α (TNF-α) were measured using the High Sensitivity Human Cytokine Milliplex™ MAP kit (MPXHCYTO-60K) (Millipore Corp., Billerica, MA, USA) and collected by flow cytometry (Luminex-100 v.2.3, Luminex Corporation, Austin, TX, USA). CRP levels were quantified by immunoturbidimetry (AU 2700, Olimpus, Rungis, France).

#### 2.3.4. Adolescents Mediterranean Diet Score (aMDS)

Dietary intake was obtained with two non-consecutive 24-h dietary recalls via the HELENA-DIAT software (Dietary Assessment Tool; Ghent University, Ghent, Belgium) within a period of two weeks, comprising weekdays and weekend-days (except from Fridays and Saturdays), though not necessarily including a week and weekend-day for each individual [31]. Adolescents completed the program autonomously in the computer classroom during school time and dietary intake was referred to the day before the interview; therefore, no information on Fridays and Saturdays was available. Fieldworkers were present to give assistance if necessary.

The adherence to the Mediterranean dietary pattern was assessed by an adapted version of the traditional Mediterranean diet score (MDS) [32]. The MDS includes 9 components (vegetables, fruits and nuts, legumes, cereals, fish, monounsaturated fat/saturated fat ratio, dairy product, meat and poultry and wine); each component is assigned a score of 0 or 1 using the gender-specific medians as cut-off values (below and above, respectively). Dairy products and meat (including poultry), as detrimental components, are reverse scored, as well as the alcohol intake above the acceptable range. In this study, we modified the MDS to adapt it for adolescents (aMDS). The aMDS was calculated for each day and a mean of the daily scores was taken as the participant’s global score. There were two dietary factors modified from the original MDS according to previously published work [33]. The alcohol component was removed because ethanol consumption is not recommended for children and adolescents. We also used age- and sex-specific median food intakes of the study’s individuals as a cut-off value for each component. The possible range of scores was 0–8, with a higher score indicating higher adherence to Mediterranean diet. Based on these results, participants were categorized into two groups: low (<4 points) and high (≥4 points) adherence.

#### 2.3.5. Statistical Analyses

Descriptive characteristics are presented as mean (SD) and percentages for continuous and categorical variables, respectively. All immune variables were checked for normality of distribution by the Kolmogorov-Smirnov test; logarithmical transformation was used to achieve normality when needed. Analysis of variance (ANOVA) was performed in order to investigate association between cortisol biomarkers and aMDS. A multiple linear regression was applied to investigate the association between cortisol biomarkers and inflammatory cytokines. The model was adjusted for age, gender, parental education and BMI z-score. Then, aMDS was included in the model and finally, the analyses were performed separately by lower and higher aMDS groups. Results are presented as coefficients and *p* values. Analyses were carried out in SPSS 22 and statistical significance was set at *p* ≤ 0.05.

## 3. Results

### 3.1. Characteristics of the Population

The social demographic characteristics of the study population divided by gender is presented in Table 1. There was significantly higher parental education level among boys (68.6% vs. 47.4% university degree; *p* = 0.006). There were no other differences between the sexes in relation to age, pubertal stage, BMI or adherence to aMDS (Table 1).

### 3.2. Inflammatory Markers, Cortisol and Adherence to the Mediterranean Diet

Increased levels of TNF-α were significantly associated with increased levels of cortisol when adjusted by age, gender, parental education and BMI (*B* = 11.882, *p* = 0.001). There was no association with other inflammatory markers. Cortisol biomarkers were inversely associated with adherence to the Mediterranean diet (overall ANOVA *F* = 5.592, *p* = 0.004, *B* = −1.023, *p* = 0.002).

Table 2 shows the unadjusted levels of inflammatory biomarkers according to adherence to Mediterranean diet. Lower IL-1, IL-2, IL-6 and TNF-α biomarkers were observed among adolescents with higher aMDS. 

### 3.3. Effect of Mediterranean Diet Adherence on Cortisol and Inflammatory Markers

The association between TNF-α and cortisol biomarkers was reduced to non-significant when we adjusted for adherence to aMDS (*F* = 1.08, *p* = 0.362, *B* = 6.118, *p* = 0.139). When we divided the groups in lower and higher adherence to aMDS the association between cortisol and TNF-α only observed in those who lower adherence to the aMDS (*B* = 14.59; *p* = 0.028) (Table 3).

## 4. Discussion

In the present study we have investigated the effect of adherence to the Mediterranean diet on the association between cortisol, a main end product of the stress system and inflammation in adolescents. We have found that cortisol was associated to TNF levels and that higher adherence to the Mediterranean diet counteracted this association. These results suggest that a healthy dietary patterns may exert a protective effect on the association between stress and inflammation.

The Mediterranean diet is a healthy dietary pattern and is defined by high intake of olive oil, fruits and vegetables, fish and seafood and a low intake of dairy and meat [34]. In general, adherence to the Mediterranean diet is associated with a reduced risk of several chronic diseases [34]. Despite its benefits, even in the Mediterranean region, adherence to the Mediterranean diet varies [35]. The effect of the Mediterranean diet on chronic diseases may be associated with the reduction of the inflammatory state, mainly measured by CRP and IL-6 markers in epidemiological adult cohorts [36] and adolescents [33]. Similar to such other studies, we also found adherence to the Mediterranean Diet associated to decreased levels of IL-6, IL-1, IL-2 and TNF-α. This study also agrees to previous HELENA analyses that evaluated Mediterranean diet food groups separately and found that IL-6 was negatively associated with cereal and roots and positively with dairy products consumption. There was also a positive association between IL-6 and both pulses and monounsaturated/saturated fat ratio [15]. Moreover, in a recent study conducted by Sureda et al. [12], the authors observed that among adolescent girls, higher adherence to Mediterranean diet was associated with lower levels of CRP. Although not the aim of our study, further studies are needed to clarify whether the same inflammatory marker are associated to Mediterranean diet in adults or adolescents.

Our study is also in accordance to a previous HELENA study which found that adolescents’ perceived stress is a significant independent negative predictor of a healthy dietary pattern, as assessed by a diet quality index [37]. In older people, higher depressive symptoms is associated with increased IL-6 levels in those who did not adhere to Mediterranean diet, while in those who adhered had lower IL-6 levels [13]. TNF-α is a pro-inflammatory cytokine upstream to IL-6 and CRP is known to induce IL-6 levels. Although this protective role of Mediterranean diet may be due to combined properties of its components, some mechanistic hypotheses have been suggested from the key elements of the Mediterranean diet. Diets with higher n-6: n-3 PUFA ratios, for example, may enhance risk of both depression and inflammatory diseases, characterized by higher levels of IL-6 and TNF-α [38].

Some limitations of this study need to be considered. This study is cross-sectional and therefore we could not infer causality. We did not have smoking on our dataset and smoking may influence levels of inflammatory biomarkers. Nevertheless, to the best of our knowledge, this is the first study to evaluate stress biomarker, inflammation and adherence to the Mediterranean diet in adolescents and suggests that the Mediterranean diet may counteract stress-induced inflammation.

## 5. Conclusions

Higher adherence to the Mediterranean diet may counteract the effect of stress-induced inflammation and decrease risk of future mental health.

## Figures and Tables

**Table 1:** Characteristics of the adolescent population studied.

Characteristics	Total	Boys	Girls	*p* *
	*n* = 242	*n* = 105	*n* = 137	
Age, years; mean (Standard Deviation, SD)	14.4 (1.1)	14.6 (1.1)	14.8(1.1)	0.222
Pubertal stage; %				0.091
Stage I	0.8	1.9	0.1	
Stage II	6.3	7.7	5.1	
Stage III	23.3	18.3	27.2	
Stage IV	35.4	31.7	38.2	
Stage V	34.2	40.4	29.4	
Parental education; %				0.006
Lower education-Higher secondary	43.4	31.4	52.6	
University degree	56.6	68.6	47.4	
BMI categories; %				0.485
Underweight/Normal weight	85.9	85.7	86.2	
Overweight/Obesity	14.1	14.3	13.8	
aMDS; mean (SD)	4.2 (1.5)	4.3 (1.5)	4.1 (1.5)	0.257

* *p*-values of independent samples *t*-tests for continuous variables and Pearson’s chi-square tests for categorical variables. BMI, Body mass index; aMDS, adolescent Mediterranean diet score ranging 0–8, with a higher score indicating higher adherence to Mediterranean diet.

**Table 2:** Inflammatory markers in adolescents with low and high adherence to the Mediterranean diet.

Inflammatory Cytokines (mean, SD)	Low Mediterranean Diet Adherence *n* = 155	High Mediterranean Diet Adherence *n* = 87	*p*-Value *
IL-1 (pg/mL)	1.1 (2.3)	0.6 (1.0)	0.022
IL-2 (pg/mL)	7.4 (13.4)	4.8 (6.8)	0.049
IL-4 (pg/mL)	153.2 (300.5)	98.3 (214.0)	0.101
IL-6 (pg/mL)	24.2 (35.7)	15.8 (20.7)	0.020
TNF-α (pg/mL)	6.7 (3.8)	5.7 (2.4)	0.013
CRP (mg/L)	0.6 (0.9)	0.8 (1.2)	0.377

* *p*-values of independent samples *t*-tests. Mediterranean diet adherence groups: Lower than 4 meaning low adherence and equal to 4 or higher meaning high adherence to adolescent Mediterranean diet score (aMDS), IL, interleukin; TNF-α, tumor necrosis factor-α; CRP, C-reactive protein. Interleukins were logarithmical transformed to achieve normality.

**Table 3:** Association between cortisol biomarkers (nmol/L) and inflammatory cytokines, according to adherence to adolescent Mediterranean diet score (aMDS).

	Cortisol Biomarkers (nmol/L)			
	Low Mediterranean Diet Adherence *n* = 155		High Mediterranean Diet Adherence *n* = 87	
	*B*	*p*-Value	*B*	*p*-Value
IL-1 (pg/mL)	0.831	0.962	−2.9	0.737
IL-2 (pg/mL)	−0.323	0.931	3.189	0.240
IL-4 (pg/mL)	−0.308	0.760	0.064	0.921
IL-6 (pg/mL)	−0.714	0.718	1.001	0.419
IL-7 (pg/mL)	0.569	0.851	0.892	0.537
TNF-α (pg/mL)	14.59	0.028	7.853	0.131
CRP (mg/L)	75.06	0.383	−63.73	0.065

Mediterranean diet adherence: Lower than 4 meaning low adherence and equal a 4 or higher meaning high adherence to adolescent Mediterranean diet score (aMDS); *B* = standardized beta. Subjects with CRP > 10 mg/mL (acute inflammation) were excluded from the analysis. IL, interleukin; Interleukins were logarithmical transformed to achieve normality. Linear regression analysis adjusted for age, gender, parental education and body mass index categories.
